# Interactions of plasma-activated water with biofilms: inactivation, dispersal effects and mechanisms of action

**DOI:** 10.1038/s41522-020-00180-6

**Published:** 2021-01-27

**Authors:** Anne Mai-Prochnow, Renwu Zhou, Tianqi Zhang, Kostya (Ken) Ostrikov, Sudarsan Mugunthan, Scott A. Rice, Patrick J. Cullen

**Affiliations:** 1 School of Chemical and Biomolecular Engineering, The University of Sydney, Darlington, NSW 2006 Australia; 2 School of Chemistry and Physics, Queensland University of Technology, Brisbane, QLD 4000 Australia; 3 The Singapore Centre for Environmental Life Sciences Engineering, Nanyang Technological University, Singapore, 639798 Singapore; 4 The School of Biological Sciences, Nanyang Technological University, Singapore, 639798 Singapore; 5 The ithree Institute, The University of Technology Sydney, Sydney, NSW 2007 Australia

**Keywords:** Biofilms, Applied microbiology

## Abstract

Biofilms have several characteristics that ensure their survival in a range of adverse environmental conditions, including high cell numbers, close cell proximity to allow easy genetic exchange (e.g., for resistance genes), cell communication and protection through the production of an exopolysaccharide matrix. Together, these characteristics make it difficult to kill undesirable biofilms, despite the many studies aimed at improving the removal of biofilms. An elimination method that is safe, easy to deliver in physically complex environments and not prone to microbial resistance is highly desired. Cold atmospheric plasma, a lightning-like state generated from air or other gases with a high voltage can be used to make plasma-activated water (PAW) that contains many active species and radicals that have antimicrobial activity. Recent studies have shown the potential for PAW to be used for biofilm elimination without causing the bacteria to develop significant resistance. However, the precise mode of action is still the subject of debate. This review discusses the formation of PAW generated species and their impacts on biofilms. A focus is placed on the diffusion of reactive species into biofilms, the formation of gradients and the resulting interaction with the biofilm matrix and specific biofilm components. Such an understanding will provide significant benefits for tackling the ubiquitous problem of biofilm contamination in food, water and medical areas.

## Introduction

Biofilms are complex communities of microbial cells (bacteria, archaea, and fungi) that are attached to a living or non-living surface and are encased within self-produced extracellular polymeric substances (EPS)^1^. In some cases, the biofilms are suspended in an aqueous environment, where they are not attached to a substratum, but rather each other (i.e., activated sludges). In natural environments, bacterial cells tend to clump as multicellular aggregates^2,3^ and in chronic infections cells can also form small aggregates that are embedded in host material^4^. These aggregates have similar characteristics to surface-attached biofilms. Most bacteria opt to grow as biofilms rather than planktonic cells because of the advantages of a community lifestyle, including the resistance offered by the EPS, enhanced cell-to-cell communication, gene transfer and sharing of metabolic workloads. The formation of biofilms has been linked to changes in gene expression that lead to the expression of specific adhesins, the production of the extracellular matrix, as well as genes involved in protection from stress and antimicrobial compounds.

Despite being responsible for the expression of various genes involved in biofilm formation, microorganisms make up only 5–35% of the total biofilm volume with the remaining volume being water and extracellular polymeric substances (EPS)^5^. Many of the unique properties of biofilms are linked to the EPS. This is because the EPS material restricts the mobility of biofilm cells, keeping them in close proximity which results in intense interactions, including cell–cell communication, horizontal gene transfer and the formation of synergistic micro consortia^6^. The biofilm EPS varies greatly between organisms and can include a range of biopolymers such as proteins, polysaccharides, DNA, RNA, lipids, surfactants and water that is important for the control of nutrient flow in the biofilm^7^. The matrix has been called ‘the dark matter of biofilms’ because of the large range of matrix biopolymers and the difficulty in their analyses^6^. Its diverse composition makes it challenging to understand its exact nature and thus simple eradication strategies are not always apparent. The thickness of the EPS matrix can be several µm while the total thickness of the biofilm varies from 50 to 400 µm^8^. It has been suggested that the ratio of EPS components to cell biovolume varies markedly throughout biofilm development with a ratio of >1 in the beginning and a ratio of <1 in more mature stages^9^. The EPS layer around biofilms is associated with the spatial organisation of cells within the biofilm, as well as physiological stratification based on the formation of gradients of nutrients, waste-products, signalling molecules etc. This physiological heterogeneity results in cells that are actively dividing, as we as cells that show reduced growth rates and the formation of persister cells, all of which contribute to tolerance to antimicrobials^10^. As a result, the effectiveness of conventional antibiotics, biocides and usual immune clearance is severely limited, and consequently, biofilms are often implicated in chronic and persistent infections^11^. Indeed, it is estimated that 65% of microbial infections and 80% of chronic infections are associated with biofilm formation^12^. While there is a considerable amount of information on the genetic pathways that drive biofilm formation, a major concern is the poor understanding of structural complexities and distribution of EPS in response to stresses. For example, existing methods have failed to decode the nature of the interaction of antibiotics to EPS on administration.

In addition to understanding the complexities of the EPS matrix biofilm behaviour and resistance is also governed by environmental surrounding where biofilms exist as mixed-species communities rather than a single species. These mixed communities interact with each other and could establish cooperative or competitive behaviour, resulting in an increase or a decrease in antimicrobial resistance. Mixed biofilm consortia have been found to contaminate food and water surfaces and are often highly resistant to removal treatments. For example, pathogens like *Listeria monocytogenes* and *Salmonella* Typhimurium can exist as a biofilm community and cross-contaminate food surfaces^13^ and multi drug-resistant pathogens like *Pseudomonas aeruginosa* and *Staphylococcus aureus* colonise medical devices causing a higher risk of chronic infections^14^. This has prompted renewed interest in alternatives to conventional antibiotics with multiple inactivation mechanism that would attack and destabilise different biofilm communities and its matrix components universally thereby making the biofilms more susceptible to antimicrobials. One such promising alternative is cold plasma and plasma-activated water (PAW).

Plasma is referred to as the fourth state of matter where increases in the material’s energy levels convert its state from solid to liquid to gas and ultimately to an ionised state of the gas, ‘plasma’, which exhibits a highly reactive environment. Plasma technology can be customised in a unique way according to different needs due to the flexibility in availability of the input gas and the plasma source design. Plasma activated liquid (PAL) is derived from the interaction of atmospheric plasma with liquids (e.g., growth media or water). Most commonly PAL is made from water and termed plasma-activated water (PAW). It has been shown to be a promising biocidal agent due to the active species formed in the plasma gas phase leading to the formation of transient active species in the liquid. For air plasmas, the resulting PAW contains high levels of reactive oxygen and nitrogen species (RONS). Studies on PAW reactivity have shown the presence of different reactive chemicals, including hydroxyl radicals (OH•), hydrogen peroxide (H_2_O_2_), ozone (O_3_), superoxide (O_2_^−^), nitric oxide (NO•) and peroxynitrite (ONOOH) and their role in bacterial inactivation^15^. Moreover, PAW is often acidic, and the lower pH may contribute to its antimicrobial effect^16^.

PAW has evolved as a technology for microbial decontamination especially on food surfaces, and medical applications such as wound healing^17^. However, inactivation of sessile biofilm bacteria (both Gram negative and positive) by PAW is a relatively new field of study^18–20^. For example, Pan et al. used PAW on dental waterlines to inactivate *Enterococcus faecalis* biofilms. The results indicated the inactivation of the bacteria within 5 min of the treatment due to the presence of RONS within the PAW^21^. However, a detailed understanding of the mode of action of PAW and in particular its interactions with the EPS matrix and other biofilm components is necessary and thus is the focus of this review. A potential stress-induced adaptation of the biofilm that may limit the efficacy of PAW and impact on the biophysical structure of the matrix that has important applications for the removal of the biofilm from surfaces is also discussed.

## How to generate PAW and regulate the aqueous RONS

It is well-know that different methods of generating PAW will lead to different amounts of reactive species, which will ultimately affect the activity of the resulting PAW for different applications^22–25^. For example, higher nitrite/nitrate concentrations in PAW might be preferred for accelerating plant growth or seed germination in agriculture^26–28^. In contrast, PAW with high levels of ROS would be beneficial for therapeutic applications, including inactivation of bacteria and viruses, biofilm removal, dentistry and cancer therapy^16,29–33^. Previous studies have shown that the RONS production strongly depends on different parameters, including the plasma power supply, applied voltage, treatment time, discharge frequency, type of feeding gas, electrode configuration, the volume of solution, the distance between the electrode and the liquid surface^25,34–36^. It was recently shown that an increase in discharge frequency led to a higher amount of key reactive species, including hydrogen peroxide, nitrite, nitrate and ozone. This, in turn, led to a higher reduction in viable bacterial cells^37^. Generally, the design of a PAW reactor will influence the production of specific ROS or RNS. The formation pathways of the desired species have to be taken into account. For example, the RNS species NO is produced by a reaction of N from gas and O from water. Thus, it is generated by a combination of one radical/atom from the plasma gas phase and one radical/atom from the water, and the reaction happens at the interface. In order to maximise its production N_2_ should be dissociated more effectively, so the plasma discharge design should be aimed for enhancing the reaction to dissociate N_2_ in the gas phase. This could be achieved by designing the electrode to have a larger contact area with the water.

Depending on the location of the cold atmospheric plasma discharge in relation to the liquid, the plasma-liquid interactions can be classified into three types:

1. gas-phase plasma discharges over the surface of the aqueous solution;
2. multiphase plasma discharges where plasma is ignited in bubbles or ignited in gaseous phase but mixed with water droplets; and
3. direct plasma discharge in aqueous solution.

Different plasma-liquid interaction types will involve different diffusion processes, chemical reactions and energy transfer during these complex physicochemical processes^35,38,39^.

## Discharge over the water surface

**Fig. 1:**
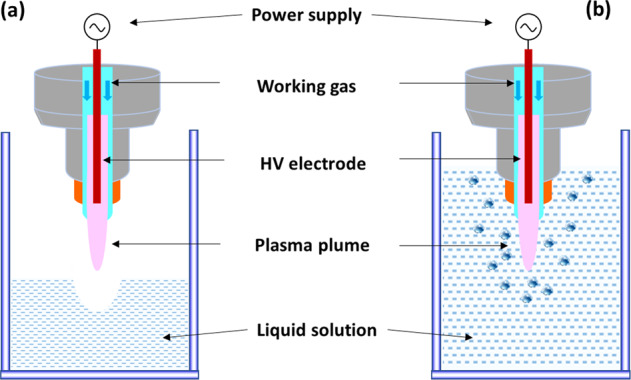
Generation of plasma-activated water. Discharge over the water surface with indirect contact of the plasma plume with the water surface (**a)** and direct contact of the plasma plume with the water surface (**b**).

A plasma discharge generated in the gaseous phase that contacts the water surface is perhaps the most commonly used configuration for PAW production. Among various electrode configurations of cold atmospheric plasma (CAP) systems, dielectric barrier discharge (DBD), plasma jets and spark or glow discharges are commonly used^40^. In a DBD system, an insulating dielectric barrier separates two electrodes where the plasma is generated. A plasma jet, a type of DBD with a central needle electrode and one outer ring electrode, or single electrode with capacitive coupling, has an additional gas flow that transports the plasma to the point of application. For spark and glow discharges a pin electrode design is used to direct the discharge to the water surface. The active constituents in PAW can form through direct contact (touch) or indirect contact (non-touch) modes of plasma-liquid interactions, as shown in Fig. 1. Under the non-touch conditions, the CAPs are generated at some distance from the water surface and there is no direct contact between the visibly gas-phase plasma and the aqueous solution. RONS are initially generated in the gaseous plasma and only long-lived RONS can subsequently dissolve and enter the solution. In the direct contact mode, apart from the accumulation of the reactive species from the gas-phase plasma, numerous additional reactive species are generated through plasma-liquid interactions in the solution. Giichiro Uchida et al. have reported that reactive oxygen and nitrogen species could be selectively produced in PAW by varying the distance of the plasma plume from the liquid surface. The ratio of NO_2_^−^ to H_2_O_2_ concentration was controlled in a wide range of 0.02–1.2 simply by increasing or decreasing the plasma-irradiation distance of the liquid surface^41^.

Selective generation and tailoring of reactive species in PAW can also be achieved through the controlling of different plasma discharge modes, plasma sources, exposure mode and working gases^41–44^. Peng Lu et al. reported comparative studies on the generation of reactive species during water exposure to open air spark discharge (SD) and glow discharge (GD) plasmas^45^. The results showed that SD-PAW contained H_2_O_2_ and NO_3_^−^, while GD-PAW contained NO_2_^−^ and NO_3_^−^, and the total reactive species concentration in SD-PAW was higher than the value of GD-PAW.

Different microwave (MW) and DBD sources were also investigated to characterise the effects of post-treatment storage on chemistry and antimicrobial properties of PAW^43^, which suggested that nitrogen‐based chemistry dominated in PAW‐MW, with high concentrations of nitrous acid decomposing to nitrite and nitrate, while H_2_O_2_ and nitrate were predominant in PAW‐DBD. Working gas is another important parameter involved in the modulation of species-specificity in plasma-treated water, as demonstrated by Girard et al., who reported that nitrogen species were dominant for a low O_2_/(O_2 _+ N_2_) percentage, while oxygen species were dominant for a higher O_2_/(O_2 _+ N_2_) percentage^44^.

Selective generation of species in PAW also facilitates its targeted applications in various fields, including plasma agriculture, bacterial inactivation and cancer therapies, where the effects of different key reactive species are actively explored. Judée et al. used DBD treated tap water for agronomy applications and suggested the formation of aqueous nitrite, nitrate, ammonium ions and hydrogen peroxide during treatment reached concentrations such that each one can induce an increase in plant growth^46^. Peng Lu et al. found that the cytotoxicity of PAW could be removed and/or enhanced by formulating their concentrations and composition through adjusting the discharge mode and time on-line during PAW generation without the addition of additional working gas or chemical scavengers^47^.

Preferential production of RONS by air and oxygen plasma in liquid was also related to bactericidal efficacy of gas-liquid plasma discharge^48^. Direct oxygen plasma treatment showed a higher inactivation effect against *S. aureus* cells due to the higher concentration of OH radicals, although the total amount of reactive species was less than that of the air plasma in the liquid. By comparison, in indirect treatment (PAW), the inactivation effect of the air-PAW was better than that of oxygen-PAW mainly due to formation of nitrogen-based reactive species such as HNO_2_, HNO_3_, ONOOH and O_2_NOOH in the liquid^48^.

## Multiphase discharges

To enhance the chemical reaction rates of the plasma-induced species interacting with liquid and to promote the mass transfer of these species from the gaseous phase to the liquid phase, multiphase plasmas are employed to improve the performance of existing plasma-based systems. This term refers to plasma that is generated in bubbles or ignited in the gaseous phase and mixed with water droplets.

**Fig. 2:**
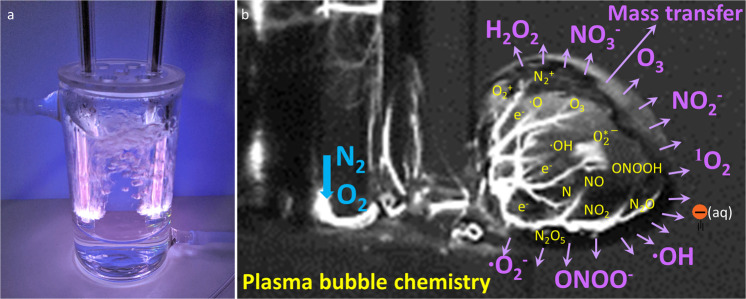
Example of plasma water reactor. Plasma bubble formation in a water reactor (**a**) and formation of reactive species in a plasma bubble (**b**). When a large number of bubbles form during plasma generation, the mass transfer of active species from the gas (bubbles) to the liquid is significantly enhanced compared to a plasma discharge at the surface of the liquid. This process leads to higher activity of the resulting PAW.

For a discharge in bubbles, a surface streamer is ignited within a forming gas bubble within a liquid volume. The discharge propagates along the gas-liquid interface (Fig. 2a), where large amounts of RONS can be generated which enhances mass transfer rates (Fig. 2b). These bubbles can act as transport vehicles to deliver RONS from the plasma to the liquid, leading to higher activity of the resulting PAW. Previous studies have demonstrated the effective chemical reaction rates from underwater plasma bubbles translating to remarkably enhanced chemical and biochemical activity for water purification, biomass conversion or biofilm reduction, owing to the high specific interfacial area, high inner pressure and relatively long residence times of plasma bubbles^29,49,50^. Wright et al. modelled the bubbly flow, interfacial mass transfer, transport of species and chemical reactions in the microbubble-DBD reactor and they suggested that the enhanced mass transfer caused a rapid increase and higher final concentrations of reactive species in the liquid phase for the same input conditions to the reactor^51^. The smaller bubble size also influenced the mixing of the liquid, and the fluid velocities induced by the airlift-loop configuration were found to be one order of magnitude larger for smaller bubbles than for larger bubbles^51^.

Plasma activated droplets, another type of plasma-treated water, are generally produced when compressed feeding gas carrying micro-sized droplets pass through the plasma discharge area (Fig. 2). Previous studies have shown that plasma-treated droplets that accumulated H_2_O_2_, NO_3_^−^ and NO_2_^−^ species and reduced the pH value demonstrated excellent antimicrobial activity against a wide range of microorganisms, including *Escherichia coli, L. monocytogenes, Salmonella* Typhimurium and *Listeria innocua*^52,53^. Compared to the conventional PAW, plasma-activated droplets can also lead to an increased surface area to volume ratio and thus enhance the transport of reactive species to facilitate the solvation of reactive species into the liquid phase. Through computer modelling, Kruszelnicki et al. reported that the liquid concentrations of stable species with high Henry’s law constant (*h*) (e.g., H_2_O_2_, HNOx) were sensitive to the radius of droplets^54^. Smaller droplets came to equilibrium with the surrounding reactive species in the gas phase in shorter times, which in turn enabled species with high *h* values to solvate into the droplet producing higher densities and lower pH. For large droplets, hydrophilic species may deplete the gas-phase inventory of RONS before liquid-phase saturation is reached, limiting the total in-liquid density for species with high *h*. On the other hand, liquid concentrations of stable species with low *h* (e.g., O_3_, N_2_O, H_2_) had a weak dependence on droplet size as droplets are quickly saturated and solvation does not deplete the gas phase^54^.

## Direct plasma discharge in aqueous solution

Direct aqueous discharge is a process where plasma is generated directly in the bulk solution. In this configuration, a high voltage electrode in the shape of a needle or plate is immersed into the liquid. Discharges inside water require a stronger electric field (1 MV/cm) than discharges in gases, because of the collisions of electrons with the denser water molecules compared to the loser molecules in air, i.e., it is harder to move through water than through air The formation of a large number of bubbles will support less energy input compared to a discharge directly into the water. The main characteristics of direct aqueous discharges include the high electron density (10^24^ m^−3^ to 10^26^ m^−3^), high gas temperature (1000–7000 K, an order of magnitude higher than in a DBD plasma), high discharge current (~several amperes). However, these features contribute to the relatively higher temperature of the treated solution, restricting the potential use of this configuration for the PAW production to non-heat-sensitive samples or the requirement for a cooling period.

## Reactive oxygen and nitrogen species in PAW

**Fig. 3:**
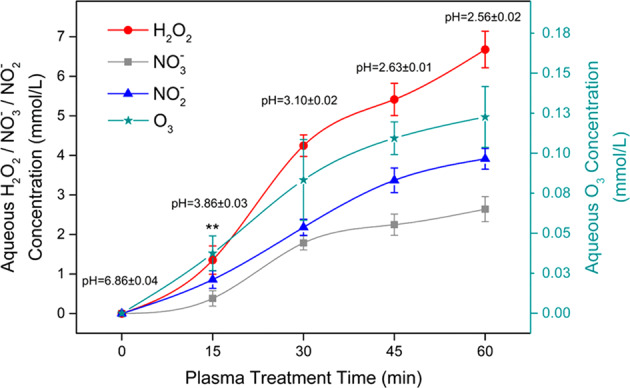
Concentrations of four kinds of representative, long‐lived aqueous reactive species (RS; H_2_O_2_, NO_3_^−^, NO_2_^−^ and O_3_) in plasma‐activated water induced by the air plasma. Each point represents the mean of nine values ± standard deviation. The significance level ***p < 0.001 for each RS concentration at every plasma inducement time, except for H_2_O_2_ at the plasma inducement time of 15 min (**p < 0.01). Reprinted with permission from ref. ^57^.

To improve and expand PAW-based applications, deducing the pathways of reactive plasma species formation in solution and quantifying the concentration of these species are important. The transfer of reactive species from plasma to the liquid consists of numerous physical and chemical processes, including molecules collision, mass transfer, liquid evaporation, sputtering, ultra-violet radiation and others^35^. It has been demonstrated by many reports that either short-lived species including; the hydroxyl radical (∙OH), nitric oxide (NO∙), superoxide (O_2_^−^), peroxynitrate (OONO_2_^−^) and peroxynitrite (ONOO^−^) with the corresponding typical half-lifetime of between 1 ns to a few seconds, or long-lived species like nitrites (NO_2_^−^), nitrates (NO_3_^−^), hydrogen peroxide (H_2_O_2_) and ozone (O_3_) with corresponding typical half-lifetimes of minutes to years contribute to the biological activity of PAW^25^. The activity of short-lived species is important when the PAW is used as an in situ treatment. Such use has been suggested when PAW is used in the fresh produce industry or for food disinfection^55,56^. The concentration of important long-lived species increases significantly with longer treatment time (Fig. 3). Although, Xu et al. showed that the observed concentration increases for H_2_O_2_, NO_3_^−^, NO_2_^−^ and O_3_ is not linear and can occur in a 2 step fashion, for example, an initial fast increase of H_2_O_2_ is followed by a slower increase after 30 min treatment^57^.

**Fig. 4:**
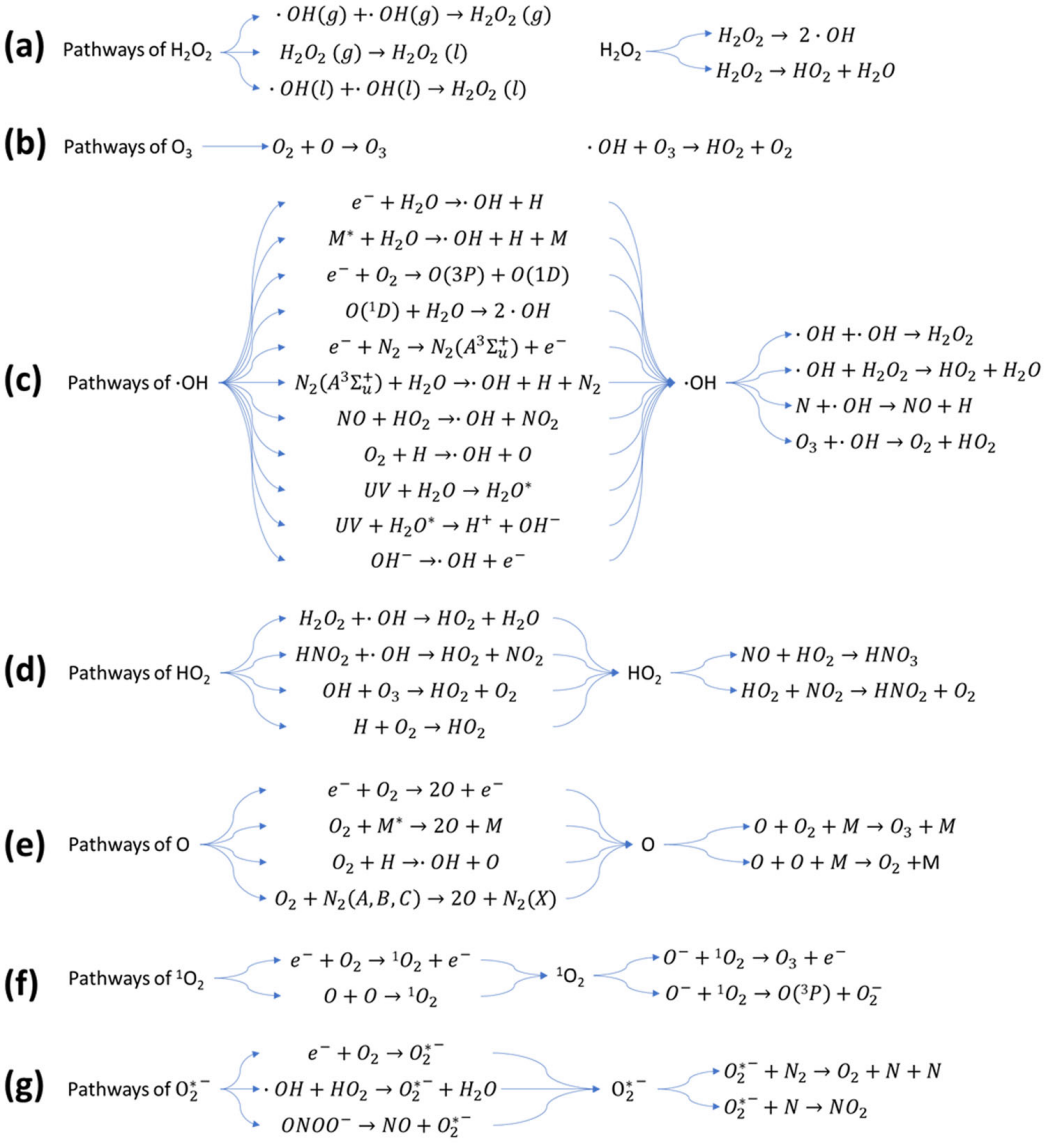
Reactive oxygen species (ROS) pathways. Pathways of: **a** H_2_O_2_, **b** O_3_, **c** ∙OH, **d** HO_2_, **e** O, **f** ^1^0_2_, **g** O_2_^*−^. Reactions show the possible formation and destruction of ROS in discharge with water.

Hydrogen peroxide (H_2_O_2_) is one of the most common long-lived reactive species in PAW, with its multifunctional activities in cell redox signalling pathways, cell oxidative stress and pathogen inactivation^58^. The formation of H_2_O_2_ is mainly from two pathways (Fig. 4a): direct transfer of H_2_O_2_ from gaseous plasmas, and direct recombination of aqueous OH radicals dissolved from the gas phase and/or reactions among other reactive species in the liquid phase^59^. The contribution of the methods depends on the plasma system used^25^. He et al. reported that in a plasma-liquid system with liquid as the cathode^59^, H_2_O_2_ was mainly formed by the combination of the dissolved OH radicals at the plasma-affected thin liquid layer, while the H_2_O_2_ formed in the gas phase and the aqueous H_2_O_2_ formation by the plasma-induced ultraviolet radiation had no contribution. However, in a conventional atmospheric-pressure plasma jet system, Qi et al. suggested that H_2_O_2_ was mainly formed by the gaseous H_2_O_2_ dissolution into liquid and the combination of aqueous OH radicals were not important for the formation of H_2_O_2_ in liquid^60^.

Ozone is considered the strongest oxidising agent when dissolved in aqueous solution^61^. It has the highest oxidation redox potential (ORP) (*E*_0_ = 2.07 V) compared to other common oxidants like hydrogen peroxide (*E*_0_ = 1.77 V), chlorine (*E*_0_ = 1.36 V) and chlorine dioxide (*E*_0_ = 1.50 V). Ozone in PAW is mainly produced through the dissolution of gaseous ozone or direct generation in liquid (Fig. 4b). Being a long-lived ROS, ozone plays a significant role in microbial inactivation and water purification. Previous reports reveal that ozone in PAW contributed to around 14% of biofilm removal^62^. But considering its relatively lower concentration in liquid than other reactive species, the contribution of ozone to the antimicrobial ability of PAW may not be very significant.

The hydroxyl radical (∙OH) is a highly reactive specie with a strong oxidising ability (*E*_0_ = 2.85 V) in aqueous environments and gas-liquid interphases^63^. Its high oxidation potential governs its short lifetime (~200 μs in the gaseous phase and few nanoseconds in liquid phase)^64^. OH radicals are mainly generated via electron impact dissociation and from secondary reactions (Fig. 4c). OH radicals may not only be effective in microbial inactivation, biofilm removal and cancer cell apoptosis when directly acting on the targets but may also play a role as an intermediate to the production of other secondary species. As ∙OH radicals are linked to the production of other short-lived and longer-lived species, they contribute to the biochemical potential of PAW in biological applications^39^.

Other ROS, including singlet oxygen (^1^O_2_), superoxide (O_2_^*−^), atomic oxygen (O) and hydrogen dioxide (HO_2_) as short-lived species act as intermediates and may also contribute to the bioactivity of PAW^25^. Their pathways and destruction are summarised in Fig. 4d–g.

**Fig. 5:**
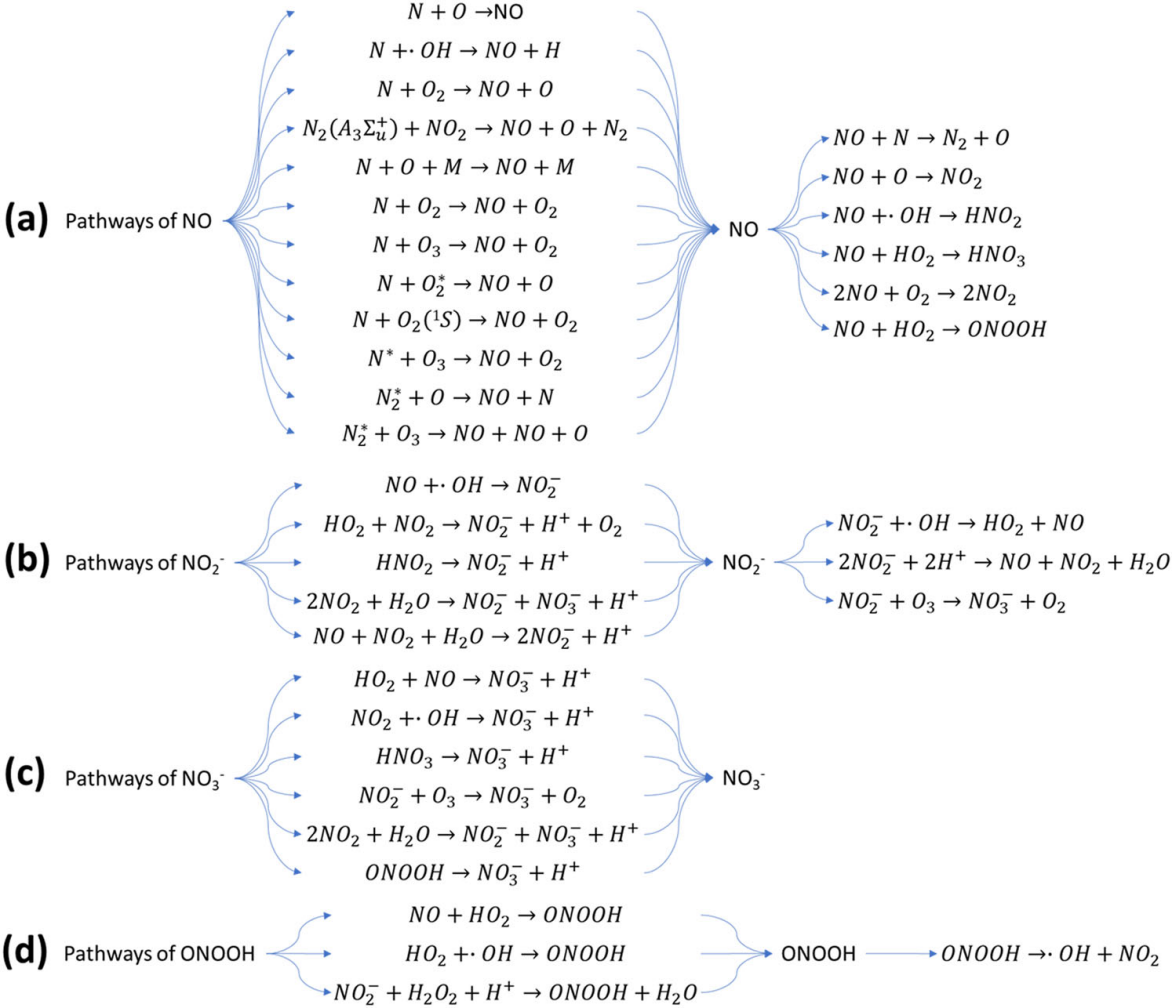
Reactive nitrogen species (RNS) pathways. Pathways of: **a** NO, **b** NO_2_^−^, **c** NO_3_^−^, **d** ONOOH. Reactions show the possible formation and destruction of RNS in discharge with water.

The reactive nitrogen species nitric oxide (NO) has been found as an omnipresent signal molecule in various life forms, and it can also be considered as a promising antimicrobial agent. As a signalling molecule, NO can penetrate cell and organelle membranes easily, resulting in the enhancement of intracellular RONS, organelle damage and cell apoptosis^65^. However, NO has a shorter lifetime when other oxidative species exist, resulting in the further production of other RNS. The pathways of NO formation are mostly formed in the gas phase, at the gas-liquid interface and in the liquid phase (Fig. 5a). Previous studies reported that aqueous NO was mostly produced from the interfacial region and the bulk, and not through solvation from the gas phase^66^. Several possible pathways of NO formation were investigated, with reactions of nitrogen species with ˙O/O_3_ in the liquid and interface the most likely ones responsible for NO amounts. Because NO is an important signalling molecule involved in wound healing or cancer, understanding its formation enables the regulation of ˙NO formation in PAW for tailored treatments.

The existence of nitrite (NO_2_^−^) and nitrate (NO_3_^−^) in PAW directly indicates the generation of RNS in the atmospheric air plasma system. NO_2_^−^ and NO_3_^−^ ions are produced in PAW through the dissolution of NO_X_ generated from the reactions of N_2_ and O_2_ in gaseous plasma discharges and the secondary reactions with other intermediates, as shown in Fig. 5b, c. Previous studies have shown that NO_2_^−^ and NO_3_^−^ as the secondary species in PAW are considered to possess strong bactericidal ability under acidified conditions^67^.

Peroxynitrite (ONOO^−^) and peroxynitrous acid (ONOOH) also play an important role in the antimicrobial ability of PAW reported by several studies^16,68^. It was reported that the chemical reactions of hydrogen peroxide and nitrite could result in the continuous production of peroxynitrite, which plays a part in bacterial inactivation^69^. Peroxynitrite could also be produced via other reactions of NO and O_2−_ radicals, NO_2_ and OH radicals, NO and HO_2_ radicals (Fig. 5d). Furthermore, the pH of PAW is also a factor which determines the destruction pathways of ONOO^−^ and ONOOH^70^. For example, when the pH is higher than 6.8 (p*K*_a_ of ONOOH), ONOO^−^ would be the dominant form, the decomposition of which is reversible and the products O_2_^−^ and NO˙ also possess high biochemical reactivity. However, under acidic conditions, most of the ONOOH would be decomposed into non-active NO_3_^−^ and H^+^ ions^16^.

## Effects of reactive species on bacteria

The antimicrobial effect of direct plasma treatment on bacteria is a combination of physical and chemical factors. There can be a drying and etching effect from a gas flow that leads to the removal of bacteria in addition to UV radiation and the consequence of all the combined reactive species^71^. When PAW is used, the main antimicrobial action often results from a low pH of the solution and the myriad of short-lived and long-lived active chemical species^25^. As mentioned above (Reactive oxygen and nitrogen species in PAW) a changing pH will affect the composition of the PAW and thus will influence the amount of certain reactive species. For example, the formation of peroxinitrite and availability of reactive decomposition products are dependent on the pH of the PAW. The pH of PAW often remains low over a longer period, whereas the composition of the solution will change over time, leading to the production of a range of RONS with different effects^72^. RONS will damage many components of the cell, including DNA, RNA, membrane components (lipids) and proteins. Because bacteria naturally encounter RONS when living aerobically, they have developed a range of protective systems such as enzymes, small proteins like thioredoxin and glutaredoxin, and molecules such as glutathione to neutralise RONS and help them survive^73^. The production of enzymes, including catalase, peroxidase and superoxide dismutase can convert harmful RONS in the cell before they cause damage^74^. Because of its action on multiple targets, it is believed that resistance to plasma is unlikely. However, low doses of repeated plasma treatment have been shown to lead to the emergence of some *P. aeruginosa* cells that have a higher tolerance to cold plasma. These cells were demonstrated to have mutations in the redox-active pigment phenazine^75^.

The role of specific components in PAW (for example H_2_O_2_, nitrate and nitrite) for cell death can be partially explained by synthesising solutions with precise concentrations of one or more of these active species and testing for resulting antibacterial activity. A study by Naïtali et al. showed that a synergistic effect of nitrite, nitrate and H_2_O_2_ and a low pH is needed to achieve a similar cell number reduction than PAW^76^. However, in many cases, there is an additional enhanced antimicrobial effect from plasma water that cannot simply be explained by the presence of the identified metastable. The effect of active species generated in PAW on bacteria depends on the bacterial species, in particular, whether the species is Gram positive or Gram negative, the physiological state (exponential or stationary growth) and the mode of growth (planktonic or biofilm).

## Role of the cell wall

Generally, Gram positive cells are more resistant to plasma treatment^77^. Gram positive cells contain a much thicker peptidoglycan layer than Gram negative cells. However, Gram negative cells possess an additional outer membrane that can give these cells different properties and plays a role in resistance to many antimicrobial agents, for example, antibiotics. For direct treatment with cold plasma, it is believed that the thicker peptidoglycan layer of Gram positive cells acts as a physical shield for plasma treatment^77^. For PAW this may be different because there is no physical etching effect and the antimicrobial action relies on the chemistry of the PAW. Indeed, a study by Hozak^78^ found that PAW was able to inhibit *S. epidermidis* (Gram positive) more efficiently than *E. coli* (Gram negative). While there was no clear explanation for this observation, the authors speculated that additional, unknown active compounds were present in their PAW that had a different effect on Gram positive and Gram negative species. In contrast, Smet et al. demonstrated that, similar to most studies using gas plasma, the use of PAW against *L. monocytogenes* (Gram positive) and *Salmonella* Typhimurium (Gram negative) led to a better inactivation of the Gram negative species. Interestingly, this was evident for both planktonic and biofilm cells^79^.

## Physiological state and mode of growth

For many antimicrobials, bacterial cells growing in the exponential phase are more susceptible than those in the stationary phase. Some evidence exists that this is also true for cold plasma inactivation. Deng et al. demonstrated that exponentially growing *E. coli* cell cultures had higher log reductions when treated with plasma compared to cells that were treated when in stationary phase^80^. However, another study demonstrated that the growth phase of *S*. Typhimurium did not significantly affect inactivation rates of cold plasma^81^. At this stage, it is unclear why the effects of PAW differ for these species and could be related to differences in the outer membrane, experimental differences or factors associated with the different growth phases.

A hallmark characteristic of biofilms is the heterogeneity of the cells. Metabolic potential varies widely among biofilm cells^82^ and activity pattern of single cells (e.g., ribosome content and cell division) differ in biofilms of the same species under the same environmental conditions^83^. Major changes in gene expression occur as a result of biofilm formation^84^. This heterogeneity will inevitably influence susceptibility to PAW and other antimicrobials.

## Interactions of PAW with biofilms

Microbial inactivation efficiency with PAW depends on the initial cell load, with higher inactivation achieved for lower population densities and a linear decline of the inactivation kinetics as a function of the log10 of the initial population^85^. Several studies observe a lower killing rate when plasma-activated water is used to disinfect biofilms compared to planktonic cells^79,85^. While the physical structure of the biofilm, e.g., high cell density and the presence of the matrix, are often implicated in the reason for a protective effect and thus a lower killing rate, the physiological state of the attached cells also plays a role. Kamgang–Youbi^85^ showed that cells that were detached from a biofilm still demonstrated higher resistance to PAW treatment than planktonic cells. This suggests that the underlying mechanism of PAW mediated inactivation depends on other, physiological cell properties.

Compared to gas plasma treatment of biofilms, the effects of reactive species on the biofilm is different when the species are delivered in a liquid form. The effect of solubility of active species that are already in a liquid (PAW) will be different from when the species are delivered in a gas phase. Moreover, while a physical etching effect (removal of biomass through plasma species bombardment) was shown to be important for biofilm inactivation when gas plasma is used^86^, both, physical and chemical characteristics need to be taken into account when analysing possible effects of the plasma species on the biofilm. In addition, treatment with PAW compared to gas plasma will avoid desiccation of the biofilm. Because of the resistance to desiccation of biofilms^87^ and the often higher tolerance to antibiotics of dry biofilms^88^ this would be an advantage for the use of PAW over gas plasma. Complex downstream chemical reactions in the PAW and the target biofilm make it difficult to match the contribution of specific active species to an effect or to determine a possible penetration depth. However, the combination of many active species and their complex interactions leading to a vast array of short-lived and long-lived species is one of the advantages of PAW. Because of the dynamic process and constant formation of different active species with antimicrobial activity, a significant killing effect is more likely.

When PAW interacts with biofilms, many effects in addition to interactions with the cells have to be taken into account. Cell type, matrix composition and thickness, biofilm structure, thickness and age are likely to play a role for the effect of PAW on biofilms. In addition, diffusion and the formation of gradients, as well as the concentrations of charged species in PAW and their resulting interactions with organic molecules present in the biofilm are important. Below we discuss the likely effect of diffusion and gradients, as well as the potential mode of action of PAW resulting from interactions with biofilm components and interference with signalling.

## Diffusion and gradient formation of reactive species in biofilms

One of the constraints that governs interactions with chemical species in biofilms is diffusion and the resulting chemical gradients. Diffusion is the main mode of transport for solutes in biofilm colonies^89^. The biofilm architecture of microcolonies, presence of EPS and water channels are often seen as contributors to the slowdown of convective transport and limit diffusion. It has been suggested that microcolonies act as molecular sieves and a diffusion coefficient is negatively correlated to the size of the colony^90^.

To determine how fast solutes such as oxygen or nitrogen diffuse into a biofilm the following formulas can be used for a flat biofilm (1) and a microcolony based biofilm (2)^91^. The formula calculates the time (t_90_) of when 90% of the bulk fluid concentration is attained at the base of a flat biofilm (1) or at the centre of a spherical microcolony (2).

1 $$t_{90} = 1.03\frac{{L^2}}{{D_e}}$$

2 $$t_{90} = 0.31\frac{{R^2}}{{D_e}}$$

*L* represents the thickness of the biofilm and *D*_e_ is the effective diffusion coefficient. For a microcolony based biofilm, *R* is the cluster radius for a spherical biofilm.

Biofilms have a very high water content of between 69–93%^92^ and thus a diffusion coefficient of water (*D*_aq_ = 2.6) can be assumed. However, the effective diffusion coefficient (*D*_e_) also depends on the temperature, viscosity and is reduced by the presence of microorganisms, EPS, abiotic particles and gas bubbles that are trapped in the biofilm^93^.

We emphasize that these are simplified calculations and do not take into account other important factors. For example, when fluid is moving adjacent to a biofilm (as could occur with PAW treatment) external mass transfer resistance will hinder diffusion^91^. In addition, reactive species that are present in the PAW will react quickly with organic matter in the biofilm and also with other reactive species. Stewart et al. demonstrated that alkaline hypochlorite (pH 11) penetrated much slower into *P. aeruginosa* and *Klebsiella pneumoniae* biofilms compared to chlorosulfamate (pH 5.5) at a similar concentration^94^. The authors concluded that the lower penetration was due to a higher reactiveness of the hypochlorite with organic matter of the biofilm whereas the transport of chlorosulfamate was not retarded.

Penetration of plasma species into samples depends on several factors, including the type of plasma, delivery mode and the gas composition to make the plasma and resulting PAW. A study by Liu et al. recently showed that active plasma species can be delivered easier into mouse skin when they are in the form of PAW compared to gas plasma^95^. Better penetration into the skin was attributed to better penetration through hair follicles, intercellular and transcellular routes. A similar penetration dependence may be envisaged for bacterial biofilms, with active species reaching cells through water channels, pores and because of the close proximity of the cells^96^.

In the case of antibiotics, it has been shown that biofilms do not simply represent a diffusion barrier to account for their higher resistance compared to planktonic cells. Instead, catalytic (enzymatic) reactions were found to be mostly responsible and diffusion and sorption only to a lesser extent^97^. Chen et al. demonstrated that PAW can diffuse freely into *E. coli* and *S. aureus* biofilms and had no notable effect on the biofilm structure. However, while there was no etching and biofilms retained the same total biomass, the PAW solutions was able to efficiently inactivate cells^18^. A study by Hozak et al. found that PAW treatment could decrease biofilm biomass of *E. coli* but led to a slight increase of biofilm biomass in *S. epidermidis*^78^. The authors speculate that the change in biomass may be related to a change in metabolic activity. For example, an increase in NO_3_^−^ ions may serve as a nitrogen source to support metabolic activity or the killing of a subpopulation can subsequently change the proportion of cells in stationary phase and increase the metabolic activity of the remaining cells. A recent study by Hathaway et al. showed the penetration of plasma generated H_2_O_2_ into biofilms of methicillin-resistant *S. aureus* (MRSA) and *P. aeruginosa* depends significantly on cell density (maturity of the biofilm). Young biofilms grown for 8 h and containing approximately 10^9^ CFU per mL reduced H_2_O_2_ transmission by about a half, whereas 24 h biofilms that had approximately 10^10^ CFU per mL prevented the detection of H_2_O_2_ almost completely^98^. It is not unexpected that thicker biofilms with more biomass for plasma species to react will present a better barrier for active plasma species and a combination with other anti-biofilm methods may be warranted.

In addition, the delivery mode of PAW to the biofilm will be important for its effectiveness. For example, a high flow of liquid during delivery will cause large shear stress on the biofilm. A high fluid shear alone (>2 Pa) leads to low-density biofilm aggregates with large interspaces^99^. A combination of high shear with other methods, including ultrasound, microbubbles and antibiotics was shown to have better biofilm removal ability compared to high shear flow alone^100^. Several studies confirmed that a physical membrane and/or EPS disruption (e.g., through high shear or ultrasound) combined with chemical treatments (e.g., antibiotics, liposomes or chemically active microbubbles) can potentiate the efficacy of the drugs^101,102^. Thus, it is feasible to apply PAW in combination with a high shear flow setting to biofilms for increased biofilm removal.

One of the characteristics of the biofilm mode of growth is the creation of concentration gradients for nutrients and metabolites. Gradients are established due to the 3-dimensional architecture of cell clusters, water channels and the surrounding matrix. Gradient formation changes localised nutrient availability and in turn, leads to heterogeneous growth of the biofilm cells. For example, the formation of distinct chemical niches within the biofilm can favour the growth and activity of some species and excludes others. This was shown for clusters of ammonia oxidisers that produce nitrite for clusters of nitrite oxidisers. The separate metabolic activities of these 2 groups led to a consumption of ammonia and oxygen near the biofilm surface and the simultaneous production and consumption of nitrite deeper underneath the surface^103,104^.

**Fig. 6:**
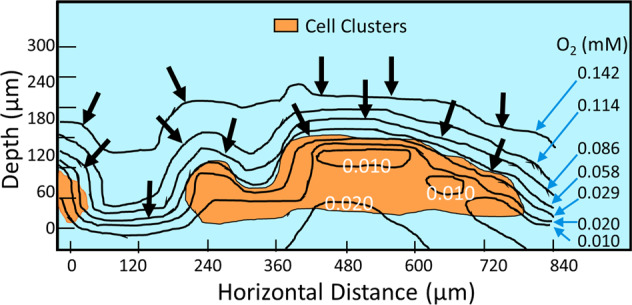
Oxygen diffusion within a microcolony. Oxygen contours and local gradients in a cross section of a 160-µm-thick biofilm. The positions of the cell clusters, shown as orange areas, were determined by microscopic observation. Numbers within the figure and on the right margin indicate the local oxygen concentrations (mM). Reprinted with permission from ref. ^106^.

The formation of gradients plays a major role in the susceptibility of biofilms to antimicrobials and are also important for the activity of PAW on biofilms. Reactive species from PAW that reach the surface of a biofilm will react and change before they reach the interior of cell clusters. Calculations considering reactiveness have been shown to be in rough agreement with experimental measurements. For oxygen, the penetration into a *P. aeruginosa* biofilm was determined to be between 50 and 90 µm^105^. Figure 6 shows a typical oxygen gradient formation for a 3 day-old biofilm demonstrating that the concentration is higher at the substratum than in the middle of the cell cluster^106^. For hydrogen peroxide, a potent species generated in PAW, the penetration depth in a *P. aeruginosa* biofilm was determined to be 30 µm by taking into account a peroxidase activity of 1 mmol per mg of total cell protein per min^91,107^. Using a catalase-deficient mutant strain Stewart et al. showed that hydrogen peroxide penetration into *P. aeruginosa* biofilms is dependent on catalase production since hydrogen peroxide was unable to penetrate or kill wild-type biofilms but did penetrate and could partially kill catalase mutant biofilms^108^. It should be noted that penetration and gradient formation is likely to be different for each chemically reactive species. Short-lived, reactive species will not be able to penetrate into the biofilm interior before they have reacted and changed into a more stable version. Moreover, calculations and measurements suggest that each of the reactive species alone may not be able to completely penetrate a biofilm to the bottom and will not be able to eliminate a thick biofilm. However, PAW comprises a cocktail of reactive species that can exert a combined effect on biofilm components and thus have the ability to eradicate biofilm cells.

## PAW-mediated mechanisms of action

The active species that are formed in PAW will react with the different biofilm components leading to several downstream effects. For example, Khosravian et al.^109^ demonstrated potential interactions of hydroxyl radicals with key biomolecule biofilm components, including alkane, alcohol, carboxylic acid, and amine. Their model suggests that organic molecules containing hydroxyl and carboxyl groups may act as trapping agents for the OH radicals in biofilms. Furthermore, OH radicals may lead to hydrogen abstraction and subsequent molecular damage. Importantly, the authors concluded that the interaction of OH radicals with biomolecules in the biofilm (polysaccharides, lipids and proteins) lead to the formation of biomolecule radicals that can initiate structural damage in the biofilm^109^.

Hydrogen peroxide is one of the active species formed in PAW. It was shown to cause DNA damage in a concentration dependant manner^110^. However, studies suggest that the direct oxidant causing the damage may be a downstream product of a secondary reaction of an iron species with hydrogen peroxide. In a Fenton reaction, ferrous iron reduces hydrogen peroxide to a reactive radical^110^. DNA damage is particularly important in a biofilm setting because extracellular DNA is a major component of EPS. Thus, hydrogen peroxide-mediated DNA damage as can occur with PAW treatment cannot only cause mutations within biofilm cells but will also affect biofilm matrix structure.

Moreover, different gene expressions and resulting different metabolic states will affect a PAW-mediated effect on the biofilm. For example, if a subpopulation of cells expresses a particular oxidative stress response (e.g., expression of catalase to neutralise hydrogen peroxide), the effect caused by reactive species produced in PAW will differ from the effect on another subpopulation of cells that does not show an oxidative stress response at that time. Given the high heterogenicity in any given biofilm a range of specific, localised reactions will take place.

Of particular interest for plasma reactions on biofilms are interactions with the biofilm matrix. The biofilm matrix is an important target for antibiofilm strategies. It is believed that a plasma discharge simply damages the chemical composition of the matrix leading to a physical release of cells. Disruption and degradation of the matrix is thought to be a major factor in biofilm dispersal^111^. Degradation of the matrix and subsequent dispersion was shown to be dependent on cyclic di-GMP and hydrolases in *P. aeruginosa*^111^. Once the matrix is disrupted biofilm cells can then detach as single cells or in larger cell clusters leaving hollow microcolony structures behind^112^. It has been demonstrated that physical disruption of the EPS in wound biofilms takes away protection and nutrition and in turn, leads to an increased rate of wound healing^113^. Moreover, a physical disturbance of the biofilm structure was able to restore antibiotic susceptibility of the otherwise resistant cells. Key antibiotic targets such as cell wall synthesis (glycopeptides) and protein synthesis (aminoglycosides) can be achieved when the biofilm starts to regenerate after disruption^114,115^. Thus, a combination therapy of PAW and subsequent antimicrobial treatment (e.g., antibiotics) may be a promising option.

Moreover, interactions with biofilm signalling should also be considered. Some of the RONS produced in PAW are important signalling molecules that affect cell communication and subsequent biofilm formation. Their interactions are therefore important when considering the effect of PAW on biofilms. ROS have been established as important signalling molecules in higher eukaryotes and more recently also shown to have regulatory functions in bacteria, especially in biofilms^112,116^. For example, intrinsically produced hydrogen peroxide can lead to localised cell death, phenotypic variation and dispersal in several bacterial species^116^.

Nitric oxide, one of the active species in PAW, is also an important biological signalling molecule^117^. NO has been shown to induce biofilm dispersal at sub-micromolar concentrations^118^. Its mode of action for biofilm dispersal has been linked to the degradation of cyclic-di-GMP by increasing phosphodiesterase activity and subsequent dispersal^119^. Interestingly, NO has been successfully used as an adjuvant with antibiotics and was able to overcome the physical barrier of the biofilm EPS matrix and potentiate the activity of antibiotics^120^. A successful controlled delivery and release of NO, as is feasible with PAW, would bring enormous benefits to therapeutic treatments of biofilms, including cystic fibrosis patients and wound infections contaminated with *P. aeruginosa*.

Moreover, bacteria communicate with each other via cell-density dependant signalling molecules (quorum sensing)^121^. Such a sophisticated communication system has many advantages for the bacteria and has been shown to be important for regulating biofilm phenotypes and virulence systems^122^. Interfering with the quorum sensing system represents an opportunity to control biofilms. Such interference would not lead to physical removal of biofilms rather disrupt certain gene expressions and make the biofilm more susceptible to subsequent removal methods.

Several studies have shown that biofilm treatment with plasma-activated water can downregulate virulence genes, including quorum sensing genes. For example, Li et al. showed that quorum sensing related virulence genes, *cylR1*, *cylA*, *gelE* and *sprE* were downregulated in the human pathogen *E. faecalis*^123^. Thus, PAW treatment may present an opportunity to disrupt quorum sensing in biofilms and therefore facilitate biofilm disruption and removal.

## Scale-up considerations

The use of PAW shows great prospects for removing biofilms from natural and innate surfaces. Thus, it is suitable to be applied in settings where microbial contamination is of concern, including in the food and medical industry. As discussed in this review, laboratory tests show a high reduction in colony forming units for a range of conditions with minimal effect on the treated surfaces. However, things that can affect the efficacy of PAW in scaled-up industry settings would be possible interactions with other components in the water, for example, traces of metals, pesticides or organic load in fresh produce wash water. These interactions may change the composition of the PAW and limit efficiency over the time course of the treatment. In addition, physical factors such as temperature will play a role. It has been shown that storage temperature greatly affects the antibacterial activity of PAW^124^. A fast decrease of nitrite and hydrogen peroxide was observed with increasing temperatures whereas the pH and oxidation reduction potential did not change significantly with temperature increases^124^.

Moreover, if PAW is to be stored before use, a loss of activity will occur that can further limit its effective use. An effective, easy to measure indicator of the current potency of PAW is needed to monitor a potential loss of activity in real-time.

PAW treatment is generally considered to be similarly cost-effective compared to other methods, such as ozone treatment and electrolysed water^25^. However, a detailed comparison between existing methods should be performed to evaluate the suitability of the uptake of the technology for real-life applications, especially for scaled-up methods.

## Conclusion/summary

**Fig. 7:**
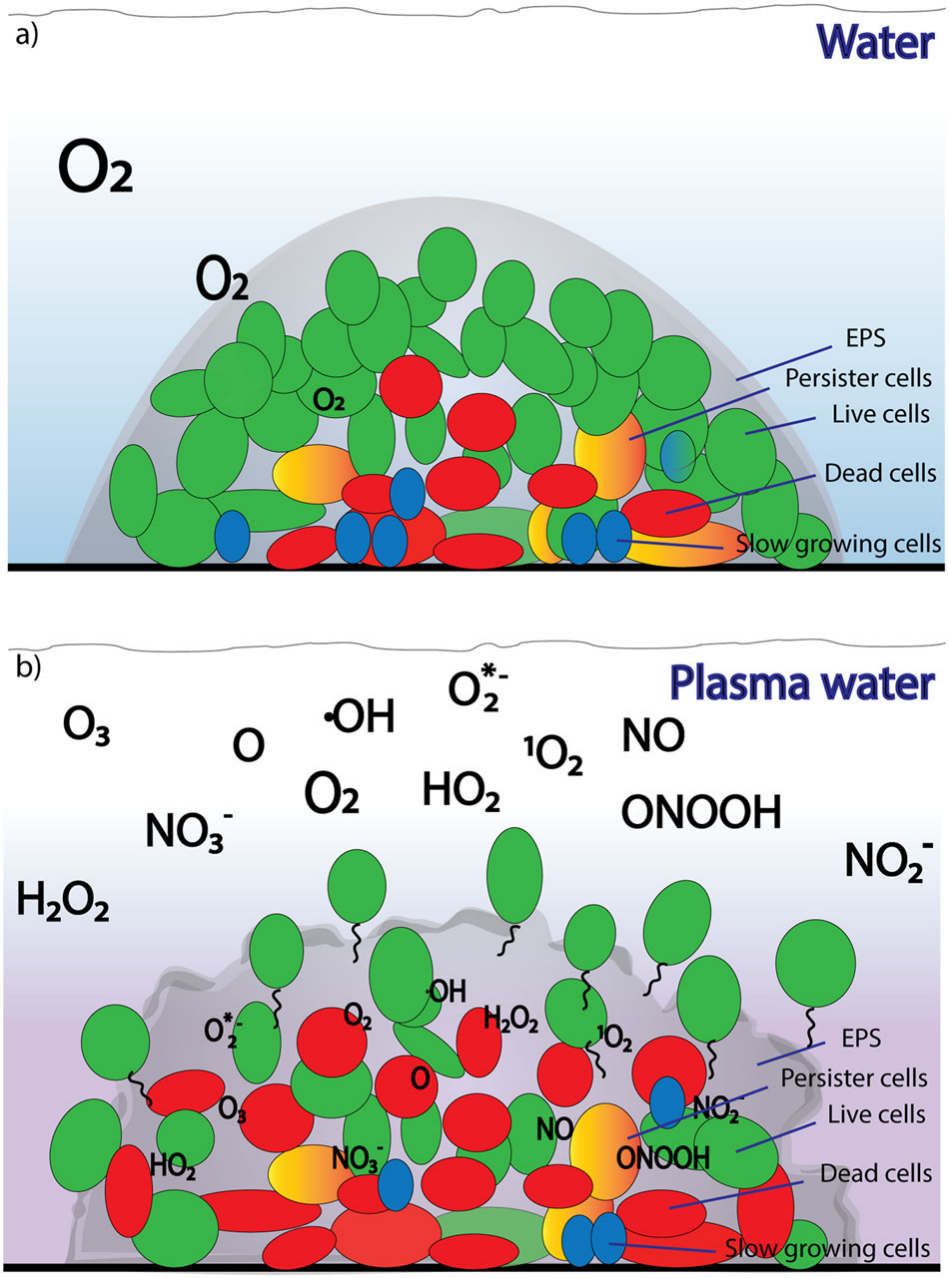
Proposed mechanism of interaction of PAW with biofilms relying on active species to disrupt the matrix leading to effective biofilm dispersal. Under non-plasma conditions an oxygen gradient will form in a microcolony (**a**). Interaction of PAW with microbial biofilms will generate a range of active RONS that can penetrate into the interior of microcolonies and kill biofilm cells. RONS will also lead to the disruption of the biofilm matrix and thus releasing cells from the biofilm interior (**b**).

Plasma-activated water represents a promising treatment for the elimination of biofilms. Depending on the generating conditions of the PAW a broad range of short-lived and long-lived RONS are produced that have a profound effect on individual cells and biofilm components and are able to disrupt the biofilm structure. Figure 7 shows a possible model for PAW biofilm interactions. The plasma generated RONS will diffuse into the biofilm and interact with the EPS matrix and other biofilm components. Additional secondary reactive species may be formed within the biofilm. Thus, biofilm treatment with PAW can lead to a disruption of the biofilm structure, such as a disruption of the matrix. This process may then release cells from the biofilm interior that can revert back to the planktonic state making them more susceptible to subsequent eradication methods.

This review critically evaluates current research data for interactions of PAW with biofilms. By outlining possible interactions of active plasma species with biofilm components it gives valuable insights into the mode of action of PAW which help to advance the application of this new technology to tackle the ubiquitous biofilm problem in food, water and medical areas.

## Data Availability

Data sharing not applicable to this article as no datasets were generated or analysed during this study.
